# Complex assessment of bone mineral density, fracture risk, vitamin D status, and bone metabolism in Hungarian systemic sclerosis patients

**DOI:** 10.1186/s13075-019-2072-y

**Published:** 2019-12-10

**Authors:** Ágnes Horváth, Edit Végh, Anita Pusztai, Zsófia Pethő, Attila Hamar, Monika Czókolyová, Harjit Pal Bhattoa, Gábor Nagy, Balázs Juhász, Katalin Hodosi, Andrea Domján, Zoltán Szekanecz, Gabriella Szücs, Szilvia Szamosi

**Affiliations:** 1 Division of Rheumatology, Department of Internal Medicine, Faculty of Medicine, University of Debrecen, Debrecen, Hungary; 2 Department of Laboratory Medicine, Faculty of Medicine, University of Debrecen, Debrecen, Hungary; 3 Department of Oncology, Faculty of Medicine, University of Debrecen, Debrecen, Hungary; 4 Faculty of Medicine, Department of Rheumatology, University of Debrecen, Nagyerdei str 98, Debrecen, 4032 Hungary

**Keywords:** Systemic sclerosis, Bone loss, Osteoporosis, DXA, pQCT, Pulmonary manifestations, Scl70

## Abstract

**Objective:**

We wished to determine bone alterations in systemic sclerosis (SSc) patients by conventional densitometry (DXA), peripheral quantitative computed tomography (pQCT), and bone biomarkers.

**Methods:**

We included 44 SSc patients and 33 age-matched healthy controls. Lumbar spine and femoral neck bone mineral density (BMD) was assessed by DXA. Volumetric BMD was measured by pQCT at the radius. FRAX, 25-hydroxyvitamin-D_3_ (25-OH-D_3_), parathyroid hormone, osteocalcin, C-terminal collagen telopeptide, and procollagen type I amino-terminal propeptide were also assessed.

**Results:**

SSc patients had lower L2–4 BMD (0.880 ± 0.108 vs. 0.996 ± 0.181 g/cm^2^; *p* = 0.019) and femoral neck (FN) BMD (0.786 ± 0.134 vs. 0.910 ± 0.090 g/cm^2^; *p* = 0.007) by DXA. In SSc vs. controls, pQCT indicated lower mean cortical (328.03 ± 103.32 vs. 487.06 ± 42.45 mg/cm^3^; *p* < 0.001) and trabecular density (150.93 ± 61.91 vs. 184.76 ± 33.03 mg/cm^3^; *p* = 0.037). Vitamin D_3_ deficiency was more common in SSc vs. controls (60.0% vs. 39.3%; *p* = 0.003). L2–4 (*p* = 0.002) and FN BMD (*p* = 0.015) positively correlated with BMI. pQCT assessments confirmed an inverse correlation between pulmonary manifestation and total (*p* = 0.024), trabecular (*p* = 0.035), and cortical density (*p* = 0.015). Anti-Scl70 positivity inversely correlated with pQCT total density (*p* = 0.015) and the presence of digital ulcers with cortical density (*p* = 0.001). We also found that vertebral and FN BMD as determined by DXA significantly correlated with pQCT total, trabecular, and cortical density (*p* < 0.05).

**Conclusion:**

The results of our study suggest that bone loss in SSc patients may be associated with lower BMI, anti-Scl70 positivity, and the presence of pulmonary manifestations and digital ulcers. Both DXA and pQCT are appropriate tools to evaluate the bone alterations in SSc patients.

## Key messages

- Bone loss in SSc patients may be associated with lower BMI and anti-Scl70.
- Osteoporosis in SSc may be connected with manifestations, such as pulmonary manifestations and digital ulcers.
- Both DXA and pQCT may be suitable to assess bone loss in SSc.

## Introduction

Systemic sclerosis (SSc) is a chronic connective tissue disease with a female predominance. There are three main contributing factors in the pathogenesis of the disease: microvascular damage, activation of fibrosis in the skin, as well as in the internal organs leading to excessive collagen deposition, and pathological immune responses resulting in T-cell activation and autoantibody production. Depending on the extent of skin involvement and specific autoantibodies, two main subsets, the limited cutaneous form (lcSSc) and the diffuse cutaneous form (dcSSc), of the disease exist. The skin and internal organ manifestations are well described and usually kept in mind during regular follow-up over the disease course. However, rather ignored musculoskeletal manifestations and osteoporosis (OP) may also have significant impact on disability and quality of life of the patients [1–3].

OP is associated with an increased risk of bone fractures due to the microarchitectural deterioration of bone tissue and a marked gradual loss of structural integrity. It has been called as “silent epidemic” as it is a growing problem and many patients are asymptomatic. There have been great advances reached in risk stratification of these patients in recent years [4]. Developing fracture risk assessment tool (FRAX) by the World Health Organization (WHO) task force in 2008 was a great leap forward the so called personalized medicine, since it provides a prediction tool for assessing an individual’s risk of fracture in order to access path for treatment decisions [5, 6]. The risk of OP is influenced by a number of factors, including peak bone mass, race, older age, family history of OP, decreased sex-hormone activity, corticosteroid (CS) use, smoking, and excessive alcohol use [4]. In addition, various chronic diseases that are either associated with chronic inflammation or affect intestinal malabsorption or vitamin D metabolism may also cause secondary bone loss [7–12]. The gold standard modality for measuring bone mineral density (BMD) is dual-energy X-ray absorptiometry (DXA). The hip and spine BMD *T*-scores derived from DXA became the basis of the WHO criteria and it is used to define specific categories: normal, osteopenic, and osteoporotic patient groups [4, 5]. Although non-DXA technologies cannot be used for conventional diagnostic classification, peripheral quantitative computed tomography (pQCT) is an established three-dimensional method to quantify BMD in the spine, proximal femur, forearm, and tibia which may predict fracture risk. The most important advantage of this technique is the unique ability to measure the cortical and trabecular bone separately [10, 13]. We have recently tested pQCT and compared this technique to DXA in rheumatoid arthritis (RA) patients [10].

Chronic autoimmune and inflammatory rheumatic diseases are well-recognized risk factors for low BMD resulting in a greater risk of fragility fractures. High prevalence of vitamin D deficiency and low BMD has been reported in patients with RA [7, 10, 11, 14], systemic lupus erythematosus (SLE) [14–16], or undifferentiated connective tissue disease (UCTD) [17]. Apart from the traditional OP risk factors, systemic inflammation plays a key role in accelerated inflammatory bone loss [7, 18, 19]. Other factors, such as physical inactivity and CS therapy, may also contribute to associated OP in inflammatory rheumatic diseases [7, 19]. Patients with SSc are also at high risk of OP and fragility fractures as a result of disease specific alterations including immobilization due to flexion contractures of the joints and muscle wasting, decreased vitamin D synthesis in the fibrotic skin, gastrointestinal malabsorption, and renal insufficiency [8, 20–25]. Although CS is not considered to have significant effect on bone density in SSc patients because of its relatively short term use, other medications, e.g., cyclophosphamide, may cause early menopause which contributes indirectly to OP in this group [8, 24]. Associations between SSc subtype, extent of skin involvement, internal organ affection, disease duration, autoantibody status, and OP have been also hypothesized, but the real role of these factors is still inconclusive [8, 24]. Further studies with longer follow-up would be required to determine whether these clinical parameters and biochemical measures are real independent effect modifiers in the development of OP in SSc patients.

Here, we investigated bone density in our SSc cohort by conventional DXA and, for the first time, peripheral QCT, in order to determine the 10-year risk of fracture using FRAX tool and to study the relationship between SSc-specific clinical and serological variables and low BMD. We also evaluated vitamin D levels and bone biomarkers and tried to find association of disease characteristics and bone parameters.

## Materials and methods

### Patients and controls

We included selected 44 SSc patients (36 women, 8 men) undergoing regular follow-ups at the outpatient clinic of the Division of Rheumatology, University of Debrecen, between March 2016 and December 2017. We used a convenience sample and included patients that had been previously referred to DXA and QCT. They had a mean age of 64.1 ± 9.0 years (range 41–82 years) and a mean disease duration of 18.0 ± 5.9 years. We selected these patients from the 252 SSc patients followed in our clinic (198 women and 54 men; mean age 61.2 ± 7.8 years; mean disease duration 16.8 ± 5.2 years). The study was approved by the local ethics committee, and all patients signed a written informed consent. All patients fulfilled the 2013 ACR/EULAR classification criteria for SSc [26]. The control group consisted of 33 age-matched healthy volunteers from the hospital staff. Patients were classified as dcSSc and lcSSc. Patients with diseases that may alter bone metabolism, such as those with endocrine disorders, chronic renal failure, liver disease, malignant hematopoietic diseases, or bone tumors, were excluded. The study was approved by the Hungarian Scientific Research Council Ethical Committee (approval No. 14804-2/2011/EKU). Written informed consent was obtained from each patient, and assessments were carried out according to the Declaration of Helsinki.

### Clinical assessment

We recorded the following clinical parameters: age, disease duration, organ involvement, menopausal status, and all relevant clinical risk factors (previous OP fractures, parental hip fracture history, BMI, alcohol use, smoking, and CS therapy) included in the FRAX by clinical charts and also by using a questionnaire. Vascular disease was defined by the presence of Raynaud’s syndrome and digital ulcers. With respect to digital ulcers, we counted lesions that were greater than 3 mm in diameter as a consequence of epithelium loss. Pulmonary manifestations were diagnosed by chest X-ray and/or high-resolution CT (HRCT) scan, and functional abnormalities were determined by pulmonary function test (PFT) including diffusing capacity for carbon monoxide (DLCO). Cardiac involvement was evaluated by echocardiography, while in cases of reasonable suspicion of pulmonary arterial hypertension (PAH), right heart catheterization (RHC) was performed. Gastrointestinal manifestation was defined by the presence of dysphagia, gastroesophageal reflux disease (GERD), and malabsorption syndrome. FRAX was assessed for all patients and controls using the online FRAX tool provided by the Centre for Metabolic Bone Diseases at Sheffield University (http://www.shef.ac.uk/FRAX/tool.jsp) [6] applying the Hungarian population reference.

### Immunolaboratory analyses and biomarkers of bone metabolism

The following serological tests were performed to detect autoantibodies: antinuclear antibodies (ANA) were determined by indirect immunofluorescence on Hep2-cells (Viro-Immun Labor-Diagnostika GmbH, Oberursel, Germany). Anticentromere (ACA; Viro-Immun Labor-Diagnostika GmbH) and anti-Scl70 antibodies (HYCOR Biomedical, Garden Grove, CA, USA) were analyzed by enzyme-linked immunosorbent assay (ELISA) in accordance with the instructions of the manufacturers. Serum levels of 25-hydroxy-vitamin D_3_ (25-OH-D_3_) were determined by high-pressure liquid chromatography (HPLC) using the Jasco HPLC system (Jasco, Tokyo, Japan) and Bio-Rad reagent kit (Bio-Rad Laboratories, Hercules, CA, USA). According to the recommendation by Dawson-Hughes et al. [27], patients with < 75 ng/ml 25-OH-D_3_ levels had hypovitaminosis. Serum parathyroid (PTH) hormone, osteocalcin (OC), C-terminal telopeptides of type I collagen (CTX), and procollagen type I amino-terminal propeptide (P1NP) were also assessed with a chemiluminescent enzyme-labeled immunometric assay (Roche Diagnostics GmbH, Mannheim, Germany).

### Bone mineral density measurements

In all SSc patients and healthy volunteers, BMD were measured by DXA (Prodigy GE Lunar, GE-Lunar Corp., Madison, Wisc., USA) at the lumbar spine (L1–L4 BMD) and femoral neck (FN BMD). BMD values were calculated in grams per square centimeter, and the results were expressed as *T*-score. Osteoporosis was defined as a lumbar spine or FN BMD *T*-score ≤ 2.5 SD according to the established WHO criteria. All BMD measurements were carried out by the same two experienced technicians.

At the time of DXA assessments, all patients and controls were also evaluated for total, trabecular, and cortical BMD of the dominant forearm by pQCT (Stratec XCT-2000, Stratec Medizintechnik GmbH, Pforzheim, Germany) as described before [10]. Data analysis was performed using the XCT6.00B software (Stratec) with measuring mask set to radius and threshold density to 269 mg/mm^3^ to define trabecular bone. BMD values are expressed as milligrams per cubic centimeter.

### Statistical analysis

Data analysis was performed using the SPSS Statistics software, version 22.0 (IBM Corps, Armonk, NY, USA). For descriptive statistics, data were presented as frequency, range, median, and mean ± standard deviation (SD). For comparisons between means, Student’s *t* test was used. For comparison between qualitative variables, independent *t* test and Mann-Whitney test were used. Correlations were determined by calculating Pearson’s correlation coefficient. Univariate and multiple regression analysis using the stepwise method was used to determine correlations and independent associations between parameters. DXA and pQCT parameters were the dependent variables and other parameters were independent variables. The *β* standardized linear coefficients showing linear correlations between two parameters were determined. The *B* (+ 95% CI) regression coefficient indicated independent association between the dependent and independent variable during changes. *p* values < 0.05 indicated statistical significance.

## Results

### Clinical characteristics of SSc patients

The main demographic and disease-specific clinical characteristics of SSc patients are summarized in Table 1. Thirty-one patients (70.4%) were menopausal, and the mean menopausal age was 46.1 ± 3.2 years. The mean duration of menopause at the time of the study was 21.5 ± 7.8 years. Only one patient (2.2%) was a long-term smoker and five patients (11.3%) reported habitual alcohol consumption. SSc patients had a mean BMI of 25.4 ± 3.9 kg/m^2^. Thirty-three patients (75%) had lcSSc, and 11 patients (25%) had dcSSc. Regarding the cumulative clinical features of SSc patients, interstitial lung disease (ILD) was most frequently seen (*n* = 35; 79.5%), followed by cardiac involvement (*n* = 29; 65.9%), dysphagia and GERD (*n* = 25; 56.8%), malabsorption syndrome (*n* = 13; 29.5%), digital ulcers (*n* = 13; 29.5%), and PAH (*n* = 3; 6.8%). None of the patients had notable renal involvement. The prevalence of ANA positivity was 75% (*n* = 33); ACA was present in seven cases (15.9%) and 11 patients (25%) were positive for anti Scl-70 antibodies.

**Table 1:** Characteristics of SSc patients

Characteristics	SSc (*n* = 44), mean ± SD or *n* (%)
Demographics and FRAX-related parameters	
Age (years)	64.1 ± 9.0
BMI (kg/m^2^)	25.4 ± 3.9
Patients in menopause	31 (70.4)
Menopausal age (years)	46.1 ± 3.2
Duration of menopause (years)	21.5 ± 7.8
Current smoking	1 (2.2)
Alcohol intake	5 (11.3)
History of fracture	19 (43.2)
Family history of hip fracture	4 (9)
Disease characteristics	
Disease duration (years)	18.0 ± 5.9
Type of disease	
Limited cutaneous	33 (75)
Diffuse cutaneous	11 (25)
Current digital ulcers	13 (29.5)
Organ involvement	
Interstitial lung disease	35 (79.5)
Cardiac involvement	29 (65.9)
Pulmonary arterial hypertension	3 (6.8)
Dysphagia and esophageal reflux disease	25 (56.8)
Malabsorption syndrome	13 (29.5)
Serological tests	
Antinuclear antibody (ANA) positivity	33 (75)
Anticentromere antibody (ACA) positivity	7 (15.9)
Anti-topoisomerase I antibody (Scl-70) positivity	11 (25)
Treatment for SSc manifestations	
Corticosteroid (short-term)	17 (38.6)
Cyclophosphamide	8 (18.1)
Other immunosuppressive drugs (MTX, AZA)	13 (29.5)
Rituximab	1 (2.2)
Treatment for OP	
Calcium supplementation	32 (72.7)
Vitamin D supplementation	32 (72.7)
Bisphosphonates	16 (36.3)
Denosumab	2 (4.5)

*Abbreviations*: *AZA* azathioprine, *BMI* body mass index, *FRAX* fracture risk assessment tool, *MTX* methotrexate. See text for further explanations

Of the 44 SSc patients, 17 (38.6%) had ever been treated with CS; however, we did not have an exact data on the cumulative CS dose. Among the 17 patients, 13 (29.5%) received CS for less than 6 months. We did not include those patients, who had been on long-term (≥ 1 year) CS therapy. Cyclophosphamide IV pulses were administered to eight SSc patients with interstitial pneumonitis or with rapidly progressive skin symptoms at doses of 750 mg/m^2^ body surface area monthly for 6–12 months. Other immunosuppressive drugs, such as oral methotrexate (MTX; 10–20 mg/week for a duration of 6–36 months) and azathioprine (AZA; 2 mg/kg) were used in 13 patients (29.5%). One patient (2.2%) received rituximab therapy for rapidly progressive skin disease and severe arthritis.

With respect to the history of fractures, 19 patients (43.2%) had altogether 23 vertebral and non-vertebral osteoporotic fractures (hip, ankle, wrist), and occurrence of hip fracture in the family history was determined in four cases (9%).

### Bone turnover metabolism and bone densitometry assessments by DXA and QCT

Table 2 shows the bone turnover markers, the 10-year probability of hip fracture, and major OP fractures (spine, forearm, hip, or shoulder) as determined by FRAX, the BMD values by DXA, prevalence of OP and osteopenia according to the WHO classification, and the pQCT measurements in patients with SSc and healthy controls.

**Table 2:** Bone turnover markers and bone status evaluated with DXA and pQCT in SSc patients and controls

Parameter	SSc (*n* = 44)	Control (*n* = 33)	*p* value
Bone turnover parameters			
Total calcium (mmol/L; mean ± SD)	2.41 ± 0.14	2.32 ± 0.11	0.001
PTH (pmol/L; mean ± SD)	5.47 ± 2.84	4.14 ± 1.38	0.008
OC (μg/L; mean ± SD)	22.22 ± 9.54	25.2 ± 7.76	0.075
CTX-I (μg/L; mean ± SD)	0.359 ± 0.193	0.28 ± 0.2	0.051
P1NP (μg/L; mean ± SD)	49.38 ± 24.47	43.2 ± 21.9	0.135
25OH D (nmol/L; mean ± SD)	53.96 ± 36.80	53.46 ± 16.35	0.47
25OH levels < 75 nmol/L (*n*; %)	32 (73%)	30 (91%)	0.06
25OH levels < 50 nmol/L (*n*; %)	26 (60%)	13 (39.3%)	0.003
FRAX			
Hip fracture (%; mean ± SD)	4.00 ± 4.36	2.31 ± 2.49	0.049
Major fracture (%; mean ± SD)	13.48 ± 8.03	9.28 ± 5.13	0.009
BMD			
Lumbar spine (L2–4; g/cm^2^; mean ± SD)	0.880 ± 0.108	0.996 ± 0.181	0.019
Femoral neck (FN; g/cm^2^; mean ± SD)	0.786 ± 0.134	0.91 ± 0.09	0.007
*T*-score			
Lumbar spine (L2–4; mean ± SD)	− 1.64 ± 1.48	− 0.50 ± 0.92	0.005
Femoral neck (FN; mean ± SD)	− 1.78 ± 1.01	− 0.44 ± 0.84	< 0.001
Femoral neck			
Osteoporosis (*n*; %)	10 (22.7%)	0 (0%)	0.004
Osteopenia (*n*; %)	23 (52.2%)	15 (45.4%)	0.009
Lumbar spine			
Osteoporosis (*n*; %)	10 (22.7%)	0 (0%)	0.004
Osteopenia (*n*; %)	16 (36.3%)	19 (57.5%)	0.106
Distal radius pQCT			
Total density (mg/cm^3^; mean ± SD)	248.42 ± 70.94	347.94 ± 40.16	< 0.001
Cortical density (mg/cm^3^; mean ± SD)	328.03 ± 103.32	487.06 ± 42.45	< 0.001
Trabecular density (mgcm^3^; mean ± SD)	150.93 ± 61.91	184.76 ± 33.03	0.037

*Abbreviations*: *BMD* bone mineral density, *CTX* C-terminal telopeptides of type 1 collagen, *D100* total volumetric BMD, *Dcort* volumetric cortical BMD, *Dtrab* volumetric trabecular BMD, *FRAX* fracture risk assessment tool, *OC* osteocalcin, *P1NP* total procollagen type I amino-terminal propeptide, *pQCT* peripheral quantitative computed tomography, *PTH* parathyroid hormone. See text for further explanations

Serum levels of calcium (2.41 ± 0.14 vs. 2.32 ± 0.11 mmol/l; *p* = 0.001) and PTH (5.47 ± 2.84 vs. 4.14 ± 1.38; *p* = 0.008) were significantly higher in SSc patients than in controls. Other bone markers, such as osteocalcin, CTX, and P1NP, did not differ significantly between the study groups. While the mean 25-OH-D_3_ levels were also comparable in SSc and controls (53.96 ± 36.80 vs. 53.46 ± 16.35 nmol/L; *p* = NS), vitamin D deficiency (25-OH-D_3_ levels < 50 nmol/L) in SSc patients (60%) was significantly more common than in controls (39.3%; *p* = 0.003). Moreover, vitamin D insufficiency (25-OH-D_3_ levels < 75 nmol/L) was rather prevalent in both groups (73% vs. 91%; *p* = 0.06) (Table 2).

The mean FRAX score for hip fractures was significantly higher in SSc patients compared to controls (4.00 ± 4.36 vs. 2.31 ± 2.49; *p* = 0.049). Similar differences were found with respect to major fractures (13.48 ± 8.03 vs. 9.28 ± 5.13; *p* = 0.009) (Table 2).

Regarding DXA, SSc patients exerted significantly lower L2–4 BMD (0.880 ± 0.108 vs. 0.996 ± 0.181 g/cm^2^; *p* = 0.019), as well as FN BMD (0.786 ± 0.134 vs. 0.910 ± 0.090 g/cm^2^; *p* = 0.007), as determined by DXA. Furthermore, L2–4 (− 1.64 ± 1.48 vs. − 0.50 ± 0.92; *p* = 0.005) and FN *T*-scores (− 1.78 ± 1.01 vs. − 0.44 ± 0.84; *p* < 0.001) were also significantly lower in SSc compared to controls. According to the WHO classification (*T*-score < 2.5), the prevalence of OP in SSc was 22.7% both at the L1–4 and FN region. In contrast, none of the control subject had OP at any measurement site.

Evaluation by pQCT indicated significantly lower mean cortical bone density in SSc patients (328.03 ± 103.32 mg/cm^3^) compared to controls (487.06 ± 42.45 mg/cm^3^; *p* < 0.001) and lower mean trabecular density in SSc (150.93 ± 61.91 mg/cm^3^) versus controls (184.76 ± 33.03 mg/cm^3^; *p* = 0.037). Similar observations were made with regards to the total volumetric BMD at the radius (D100) (248.42 ± 70.94 vs. 347.94 ± 40.16 mg/cm^3^; *p* < 0.001) (Table 2).

### Comparison of SSc subsets by qualitative variables

When assessing bone density and bone biomarkers in different SSc subsets (Table 3), women had lower FN BMD as determined by DXA, as well as lower total and cortical density as measured by pQCT compared to men (*p* < 0.05). SSc patients with pulmonary involvement (ILD) had lower pQCT total, trabecular, and cortical density vs. those without pulmonary involvement (*p* < 0.01). Patients with digital ulcer and those with anti-Scl70 positivity exerted lower pQCT total and cortical density in comparison to digital ulcer and anti-Scl70 negative patients (*p* < 0.05) (Table 3).

**Table 3:** Comparison of SSc subsets by univariable analyses

Dependent variable	Independent variable	Univariable analysis		
		*B* (95% CI)	*β*	*p* value
DXA L2–4 BMD	Age	− 0.006 (− 0.010 to − 0.002)	− 0.427	0.005
	Age at dg.	− 0.007 (− 0.013 to − 0.001)	− 0.397	0.017
	BMI	0.014 (0.005 to 0.022)	0.471	0.002
DXA FN BMD	Age	− 0.004 (− 0.007 to 0)	− 0.324	0.027
	BMI	0.009 (0.002 to 0.015)	0.346	0.027
	Female vs. male	− 0.131 (− 0.226 to − 0.036)	− 0.392	0.008
pQCT total density	Female vs. male	− 69.529 (− 115.229 to − 23.826)	− 0.382	0.004
	Pulmonary manifestation	− 51.367 (− 95.655 to − 7.078)	− 0.423	0.024
	Digital ulcer	− 56.903 (− 97.273 to − 16.532)	− 0.402	0.007
	anti Scl70+	− 50.693 (− 91.123 to − 10.263)	− 0.313	0.015
pQCT trabecular density	Pulmonary manifestation	− 48.234 (− 93.028 to − 3.440)	− 0.318	0.035
	ACA+	91.292 (47.628 to 134.956)	0.546	0.001
pQCT cortical density	Female vs. male	− 120.46 (− 193.899 to − 47.021)	− 0.455	0.002
	Pulmonary manifestation	− 82.502 (− 147.791 to − 17.212)	− 0.326	0.015
	Digital ulcer	− 92.848 (− 146.016 to − 39.680)	− 0.450	0.001
	anti Scl70+	− 94.645 (− 161.926 to − 27.365)	− 0.401	0.007

*Abbreviations*: *ACA* anti-centromere antibody, *BMD* bone mineral density, *DXA* dual-energy X-ray absorptiometry, *FN* femoral neck, *pQCT* peripheral quantitative computed tomography. See text for further explanations

Multiple linear regression analysis was performed in order to identify factors associated with low BMD assessed by DXA and QCT in SSc patients (Table 4). In our cohort, age inversely (*p* = 0.005; *p* = 0.027) and BMI positively (*p* = 0.002; *p* = 0.015) correlated with L2–4 and FN BMD, respectively, as determined by DXA. With respect to the pQCT assessments, pulmonary manifestations inversely correlated with total (*p* = 0.024), trabecular (*p* = 0.035), and cortical density (*p* = 0.015). Moreover, anti-Scl70 positivity inversely correlated with pQCT total density (*p* = 0.015) and the presence of digital ulcers with cortical density (*p* = 0.001) (Table 4).

**Table 4:** Multiple regression analysis of bone density and other parameters

Dependent variable	Independent variable	Multivariable analysis		
		*B* (95% CI)	*β*	*p* value
DXA L2–4 BMD	Age	− 0.006 (− 0.010 to − 0.002)	− 0.427	0.005
	BMI	0.014 (0.005–0.022)	0.471	0.002
DXA FN BMD	Age	− 0.004 (− 0.007 to 0)	− 0.324	0.027
	BMI	0.009 (0.002 to 0.015)	0.346	0.015
pQCT total density	Pulmonary manifestation	− 51.367 (− 95.655 to − 7.078)	− 0.295	0.024
	Scl70+	− 50.693 (− 91.123 to − 10.263)	− 0.313	0.015
pQCT trabecular density	Pulmonary manifestation	− 48.234 (− 93.028 to − 3.440)	− 0.318	0.035
pQCT cortical density	Pulmonary manifestation	− 82.502 (− 147.791 to − 17.212)	− 0.326	0.015
	Digital ulcer	− 92.848 (− 146.016 to − 39.680)	− 0.450	0.001

*β* standardized linear coefficient, *B (+ 95% CI)* regression coefficient, *BMD* bone mineral density, *BMI* body mass index, *DXA* dual-energy X-ray absorptiometry, *FN* femoral neck, *L* lumbar, *pQCT* peripheral quantitative computed tomography. See text for further explanations

Among the 44 SSc patients, 19 had OP and 25 did not. When comparing OP and non-OP patients, those with OP were significantly older (69.4 ± 10.4 vs. 61.6 ± 10.1 years; *p* = 0.016), had lower BMI (23.0 ± 3.5 vs. 27.1 ± 5.0 kg/m^2^; *p* = 0.007), and had higher FN FRAX value (6.07 ± 3.80 vs. 2.54 ± 4.20; *p* < 0.001) than those without OP (data not shown).

### Correlations between densitometry and pQCT bone density values in SSc patients

**Fig. 1:**
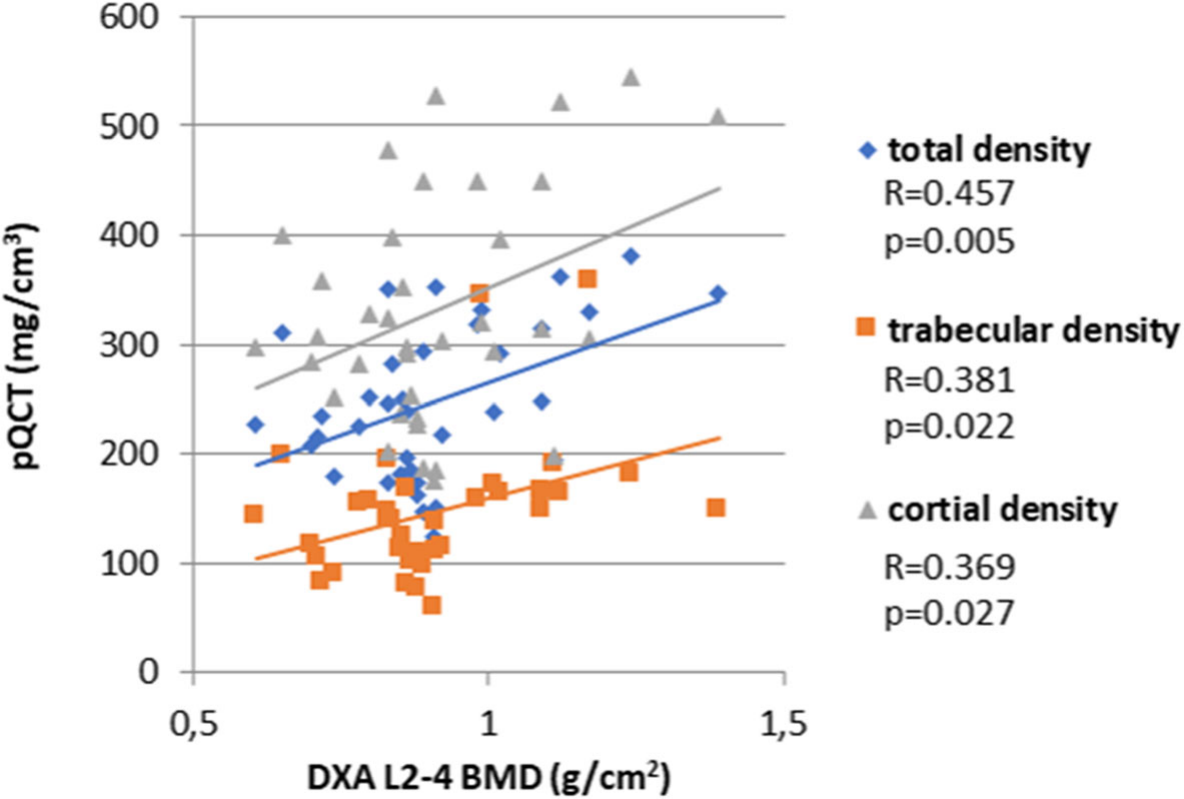
Significant correlations between lumbar spine DXA and pQCT bone density values

**Fig. 2:**
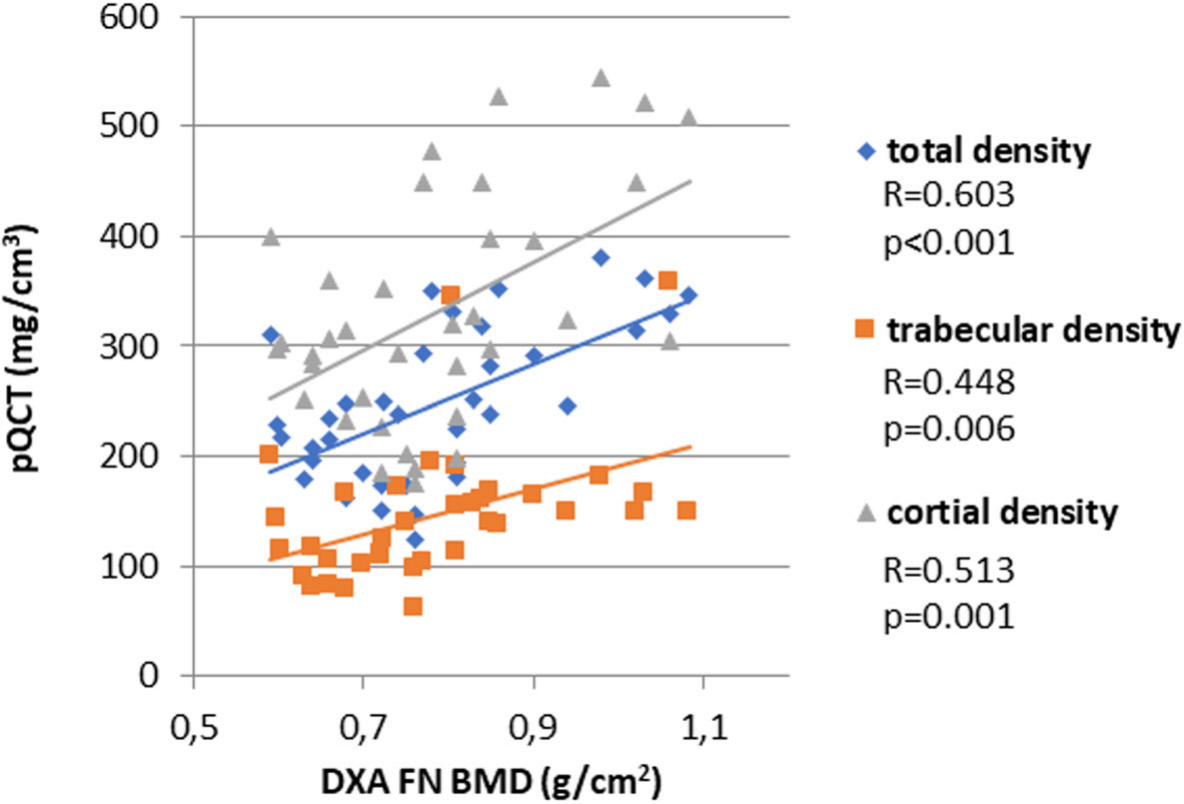
Significant correlations between femoral neck DXA and pQCT bone density values

Figure 1 presents the correlations between DXA and pQCT parameters. The diagram shows that both vertebral and FN BMD as determined by DXA significantly correlated with pQCT total, trabecular, and cortical density (*p* < 0.05) (Fig. 2).

## Discussion

OP has been associated with a number of inflammatory rheumatic diseases including SSc [8, 12, 21, 25]. To our knowledge, this may be the most complex study applying standard DXA, forearm pQCT, and FRAX, as well as bone biomarkers in order to evaluate bone density and bone turnover in SSc patients in comparison to healthy controls. With respect to clinical features, 70% of SSc patients were postmenopausal. In the past, more than 40% of the patients had fragility fractures. Menopause plays an important role in the acceleration of bone loss. Others reported significantly greater proportion of SSc patients in menopause than in controls, which occurred significantly earlier in that group [28]. Some authors also noted a longer duration of menopause in SSc patients [29]. In our study, the frequency of menopause was not significantly different in SSc patients and age-matched control group.

When the bone status of SSc patients was compared to that of controls, vitamin D deficiency was more prevalent in SSc versus controls. We [14, 22] and others [30–33] have also found D hypovitaminosis in SSc. Moreover, low vitamin D levels were associated with organ manifestations, such as skin involvement [22]. It has been established that vitamin D is not only involved in bone homeostasis, but also exerts important immunological effects [34, 35]. Thus, D hypovitaminosis has been implicated in the pathogenesis of various autoimmune diseases including SSc [14, 16, 22, 33, 35, 36]. Impaired VDR signaling in SSc has been reported, which, together with a decreased level of vitamin D, results in hypersensitivity of SSc fibroblasts to TGF-β signaling, leading to an uncontrolled activation of fibroblasts [31, 37]. Others found that low levels of vitamin D were associated with different disease activity markers in SSc (e.g., disease duration, ESR, CRP values, the presence of ACA, systolic PAH, pulmonary fibrosis, and nailfold capillaroscopic pattern) [30, 31, 38]. Our further analyses revealed no significant associations between low vitamin D level and any disease-specific measures, including disease subset, autoantibody positivity, or any organ manifestations.

Hip FRAX scores were also higher in SSc vs. controls. Increased fracture risk associated with various risk factors has been reported in SSc [21]; however, we found only one report on FRAX assessment in SSc. In that study, the 10-year fracture risk was higher in SSc patients with low BMD [25].

Regarding DXA, SSc patients had lower L2–4 and FN BMD and *T* score values vs. controls. Moreover, WHO-defined OP was more common is SSc. These data are in line with previous reports. Studies in the literature suggested that SSc is a risk factor for bone loss; however, the prevalence of OP was within a wide range from 3 to 51%. This wide dispersion could be attributed to the heterogeneity of the patients studied (e.g., age, gender, menopausal status, geographic location, disease subtype, organ manifestations, and CS exposure) [8, 21, 23]. Other groups also reported lower BMD at multiple sites in SSc [8, 23–25, 33].

In our cohort, total, trabecular, and cortical volumetric BMD as determined by pQCT was lower in SSc compared to controls. The difference was more pronounced in cortical BMD. To our knowledge, there has been only one study where bone pQCT was performed in SSc patients. In that study, Marot et al. [39] demonstrated significant alterations in the trabecular bone compartment; however, measures of the cortical compartment were not different in SSc patients and controls.

In our study, we also compared DXA and pQCT. The total, trabecular, and cortical density values determined by QCT all significantly correlated with L2–4 and FN BMD measured by DXA. We obtained similar results in our recent study involving RA patients compared to controls [10].

Among bone markers, we found elevated PTH levels in SSc compared to controls. Others also reported higher PTH in SSc [33]. In our cohort, OC, P1NP, and CTX levels were similar in SSc and controls. With respect to OC and P1NP, similar results were reported by others [33, 40]. Allanore et al. [41] found elevated CTX levels in 16 out of 33 patients. Moreover, we found significant associations between gastrointestinal involvement and levels of OC, P1NP, and CTX. Similar relationship has not yet been reported in SSc.

When assessing different SSc subsets, women had lower DXA FN BMD than men. In addition, SSc patients with pulmonary involvement, digital ulcers, and anti-Scl70 positivity had greater bone loss as determined by pQCT in comparison to patients without these features. Moreover, dcSSc patients compared to lcSSc patients, as well as patients with compared to without gastrointestinal manifestations, exerted higher bone turnover as indicated by bone biomarkers. In general, SSc patients with OP were older, had lower BMI, and had higher hip FRAX. Our study indicated significant, close correlations between BMD determined by DXA and volumetric bone density assessed by pQCT. Moreover, the multiple regression analysis indicated that older age and lower BMI were independently associated with lower L2–4 and FN BMD by DXA. On the other hand, disease-specific measures, pulmonary manifestations, digital ulcers, and anti-Scl70 positivity may determine pQCT volumetric density values. Our data suggested BMI as an independent influencing factor of FN and lumbar BMD by DXA. Others reported reduced lean body mass in SSc patients due to decreased physical activity, malnutrition, and CS treatment, which may contribute to a reduced BMI. They were also able to show that lower BMI was an independent risk factor for low BMD at the hip and femoral neck [42]. With regard to bone biomarkers, Allanore et al. [41] also found a correlation between CTX levels and the presence of dcSSc vs. lcSSc, higher Rodnan skin score, pulmonary manifestations, and anti-Scl70 positivity. In the abovementioned French study by Marot et al. [39], the presence of ACA and digital ulcers were associated with low BMD by DXA at all sites and trabecular density by pQCT at tibia, highlighting the suspected role of repeated vasospasm and subsequent systemic microangiopathy in the alteration and resorption of bone tissue.

As mentioned above, pQCT studies performed in SSc are scarce. In one study, the presence of digital ulcers was associated with low BMD by DXA and highlighted the suspected role of repeated vasospasm and subsequent systemic microangiopathy in the alteration and resorption of bone tissue [39].

The strength of our study is the complexity of different methods (DXA, pQCT, FRAX, bone markers) used to assess bone status in SSc. There may be some limitations, for example, the relatively small number of patients and controls included. In addition, some patients received short-term CS treatment. Also, this was a single-center study with a small number of patients, so it may not be generalizable.

In conclusion, our study suggests that the existence of OP, as well as BMD determined by traditional DXA, shows association with general features, such as age or BMI. On the other hand, volumetric bone density assessed by pQCT, as well as bone biomarkers, rather showed associations with SSc-specific features including dcSSc vs. lcSSc subtype, organ manifestations (pulmonary, digital ulcers, gastrointestinal), and anti-Scl70 positivity. These data suggest that in dcSSc patients with extensive organ involvement and anti-Scl70 positivity, pQCT and bone biomarkers may have additional value during the assessment of bone status. Larger cohort studies are needed in order to determine the real place of these techniques in determining bone status in SSc patients.

## Data Availability

N/A

## Abbreviations

25-OH-D3: 25-hydroxy-cholecalciferol
ACA: Anti-centromere antibody
ANA: Antinuclear antibody
BMD: Bone mineral density
BMI: Body mass index
CS: Corticosteroid
CTX: C-terminal telopeptide of type I collagen
DLCO: Diffusion capacity
DXA: Dual-energy X-ray absorptiometry
ELISA: Enzyme-linked immunosorbent assay
FN: Femoral neck
FRAX: Fracture risk assessment tool
GERD: Gastroesophageal reflux
HPLC: High-pressure liquid chromatography
HRCT: High-resolution CT
ILD: Interstitial lung disease
OC: Osteocalcin
OP: Osteoporosis
P1NP: Procollagen type I amino-terminal propeptide
PAH: Pulmonary arterial hypertension
PFT: Pulmonary function test
pQCT: Peripheral quantitative computed tomography
PTH: Parathyroid hormone
RA: Rheumatoid arthritis
RHC: Right heart catheterization
SLE: Systemic lupus erythematosus
SSc: Systemic sclerosis
UCTD: Undifferentiated connective tissue disease

## References

[1] Gabrielli A, Avvedimento EV, Krieg T (2009). Scleroderma. N Engl J Med.

[2] LeRoy EC, Medsger TA (2001). Criteria for the classification of early systemic sclerosis. J Rheumatol.

[3] Hudson M, Thombs BD, Steele R, Panopalis P, Newton E, Baron M (2009). Health-related quality of life in systemic sclerosis: a systematic review. Arthritis Rheum.

[4] Rachner TD, Khosla S, Hofbauer LC (2011). Osteoporosis: now and the future. Lancet.

[5] Unnanuntana A, Gladnick BP, Donnelly E, Lane JM (2010). The assessment of fracture risk. J Bone Joint Surg Am.

[6] Kanis JA, Johnell O, Oden A, Johansson H, McCloskey E (2008). FRAX and the assessment of fracture probability in men and women from the UK. Osteoporos Int.

[7] Hoes JN, Bultink IE, Lems WF (2015). Management of osteoporosis in rheumatoid arthritis patients. Expert Opin Pharmacother.

[8] Loucks J, Pope JE (2005). Osteoporosis in scleroderma. Semin Arthritis Rheum.

[9] Bernstein CN (2006). Inflammatory bowel diseases as secondary causes of osteoporosis. Curr Osteoporos Rep.

[10] Juhasz B, Gulyas K, Horvath A, Petho Z, Bhattoa HP, Vancsa A (2017). Comparison of peripheral quantitative computed tomography forearm bone density versus dxa in rheumatoid arthritis patients and controls. Osteoporos Int.

[11] Rass P, Pakozdi A, Lakatos P, Zilahi E, Sipka S, Szegedi G (2006). Vitamin d receptor gene polymorphism in rheumatoid arthritis and associated osteoporosis. Rheumatol Int.

[12] Sinigaglia L, Varenna M, Girasole G, Bianchi G (2006). Epidemiology of osteoporosis in rheumatic diseases. Rheum Dis Clin N Am.

[13] Engelke K, Adams JE, Armbrecht G, Augat P, Bogado CE, Bouxsein ML (2008). Clinical use of quantitative computed tomography and peripheral quantitative computed tomography in the management of osteoporosis in adults: the 2007 ISCD official positions. J Clin Densitom.

[14] Orbach H, Zandman-Goddard G, Amital H, Barak V, Szekanecz Z, Szucs G (2007). Novel biomarkers in autoimmune diseases: prolactin, ferritin, vitamin D, and TPA levels in autoimmune diseases. Ann N Y Acad Sci.

[15] Sun YN, Feng XY, He L, Zeng LX, Hao ZM, Lv XH (2015). Prevalence and possible risk factors of low bone mineral density in untreated female patients with systemic lupus erythematosus. Biomed Res Int.

[16] Amital H, Szekanecz Z, Szucs G, Danko K, Nagy E, Csepany T (2010). Serum concentrations of 25-OH vitamin D in patients with systemic lupus erythematosus (SLE) are inversely related to disease activity: is it time to routinely supplement patients with SLE with vitamin d?. Ann Rheum Dis.

[17] Zold E, Szodoray P, Gaal J, Kappelmayer J, Csathy L, Gyimesi E (2008). Vitamin d deficiency in undifferentiated connective tissue disease. Arthritis Res Ther.

[18] Takayanagi H (2009). Osteoimmunology and the effects of the immune system on bone. Nat Rev Rheumatol.

[19] Szentpetery A, Horvath A, Gulyas K, Petho Z, Bhattoa HP, Szanto S (2017). Effects of targeted therapies on the bone in arthritides. Autoimmun Rev.

[20] Omair MA, Pagnoux C, McDonald-Blumer H, Johnson SR (2013). Low bone density in systemic sclerosis. A systematic review. J Rheumatol.

[21] Avouac J, Koumakis E, Toth E, Meunier M, Maury E, Kahan A (2012). Increased risk of osteoporosis and fracture in women with systemic sclerosis: a comparative study with rheumatoid arthritis. Arthritis Care Res (Hoboken).

[22] Arnson Y, Amital H, Agmon-Levin N, Alon D, Sanchez-Castanon M, Lopez-Hoyos M (2011). Serum 25-OH vitamin D concentrations are linked with various clinical aspects in patients with systemic sclerosis: a retrospective cohort study and review of the literature. Autoimmun Rev.

[23] Frediani B, Baldi F, Falsetti P, Acciai C, Filippou G, Spreafico A (2004). Bone mineral density in patients with systemic sclerosis. Ann Rheum Dis.

[24] Sampaio-Barros PD, Costa-Paiva L, Filardi S, Sachetto Z, Samara AM, Marques-Neto JF (2005). Prognostic factors of low bone mineral density in systemic sclerosis. Clin Exp Rheumatol.

[25] Omair MA, McDonald-Blumer H, Johnson SR (2014). Bone disease in systemic sclerosis: outcomes and associations. Clin Exp Rheumatol.

[26] van den Hoogen F, Khanna D, Fransen J, Johnson SR, Baron M, Tyndall A (2013). 2013 classification criteria for systemic sclerosis: an American College of Rheumatology/European League against Rheumatism collaborative initiative. Ann Rheum Dis.

[27] Dawson-Hughes B, Heaney RP, Holick MF, Lips P, Meunier PJ, Vieth R (2005). Estimates of optimal vitamin D status. Osteoporos Int.

[28] La Montagna G, Vatti M, Valentini G, Tirri G (1991). Osteopenia in systemic sclerosis. Evidence of a participating role of earlier menopause. Clin Rheumatol.

[29] Ibn Yacoub Y, Amine B, Laatiris A, Wafki F, Znat F, Hajjaj-Hassouni N (2012). Bone density in Moroccan women with systemic scleroderma and its relationships with disease-related parameters and vitamin D status. Rheumatol Int.

[30] Caramaschi P, Dalla Gassa A, Ruzzenente O, Volpe A, Ravagnani V, Tinazzi I (2010). Very low levels of vitamin d in systemic sclerosis patients. Clin Rheumatol.

[31] Vacca A, Cormier C, Piras M, Mathieu A, Kahan A, Allanore Y (2009). Vitamin D deficiency and insufficiency in 2 independent cohorts of patients with systemic sclerosis. J Rheumatol.

[32] Braun-Moscovici Y, Furst DE, Markovits D, Rozin A, Clements PJ, Nahir AM (2008). Vitamin D, parathyroid hormone, and acroosteolysis in systemic sclerosis. J Rheumatol.

[33] Atteritano M, Sorbara S, Bagnato G, Miceli G, Sangari D, Morgante S (2013). Bone mineral density, bone turnover markers and fractures in patients with systemic sclerosis: a case control study. PLoS One.

[34] Yang CY, Leung PS, Adamopoulos IE, Gershwin ME (2013). The implication of vitamin D and autoimmunity: a comprehensive review. Clin Rev Allergy Immunol.

[35] Rosen Y, Daich J, Soliman I, Brathwaite E, Shoenfeld Y (2016). Vitamin D and autoimmunity. Scand J Rheumatol.

[36] Arnson Y, Amital H, Shoenfeld Y (2007). Vitamin D and autoimmunity: new aetiological and therapeutic considerations. Ann Rheum Dis.

[37] Zerr P, Vollath S, Palumbo-Zerr K, Tomcik M, Huang J, Distler A (2015). Vitamin D receptor regulates TGF-beta signalling in systemic sclerosis. Ann Rheum Dis.

[38] Vacca A, Cormier C, Mathieu A, Kahan A, Allanore Y (2011). Vitamin D levels and potential impact in systemic sclerosis. Clin Exp Rheumatol.

[39] Marot M, Valery A, Esteve E, Bens G, Muller A, Rist S (2015). Prevalence and predictive factors of osteoporosis in systemic sclerosis patients: a case-control study. Oncotarget.

[40] Alexandersson BT, Geirsson AJ, Olafsson I, Franzson L, Sigurdsson G, Gudbjornsson B (2007). Bone mineral density and bone turnover in systemic sclerosis. Laeknabladid.

[41] Allanore Y, Borderie D, Lemarechal H, Cherruau B, Ekindjian OG, Kahan A (2003). Correlation of serum collagen I carboxyterminal telopeptide concentrations with cutaneous and pulmonary involvement in systemic sclerosis. J Rheumatol.

[42] Mok CC, Chan PT, Chan KL, Ma KM (2013). Prevalence and risk factors of low bone mineral density in Chinese patients with systemic sclerosis: a case-control study. Rheumatology (Oxford).

